# Numerical Simulation of Three-Dimensional Free Surface Flows Using the K–BKZ–PSM Integral Constitutive Equation †

**DOI:** 10.3390/polym15183705

**Published:** 2023-09-08

**Authors:** Juliana Bertoco, Antonio Castelo, Luís L. Ferrás, Célio Fernandes

**Affiliations:** 1 Center for Mathematics, Computing and Cognition — CMCC, Federal University of ABC — UFABC, Santo André 09210-580, Brazil; 2 Department of Applied Mathematics and Statistics, University of São Paulo — USP, São Carlos 13566-590, Brazil; castelo@icmc.usp.br; 3 Department of Mechanical Engineering (Section of Mathematics), Faculty of Engineering of the University of Porto — FEUP, 4200-465 Porto, Portugal; lferras@fe.up.pt; 4 Center of Mathematics — CMAT, University of Minho, 4800-058 Guimarães, Portugal; cbpf@fe.up.pt; 5 Transport Phenomena Research Centre — CEFT, Faculty of Engineering at University of Porto — FEUP, 4200-465 Porto, Portugal

**Keywords:** K–BKZ, PSM, free surface, Boger fluids, finite difference

## Abstract

This work introduces a novel numerical method designed to address three-dimensional unsteady free surface flows incorporating integral viscoelastic constitutive equations, specifically the K–BKZ–PSM (Kaye–Bernstein, Kearsley, Zapas–Papanastasiou, Scriven, Macosko) model. The new proposed methodology employs a second-order finite difference approach along with the deformation fields method to solve the integral constitutive equation and the marker particle method (known as marker-and-cell) to accurately capture the evolution of the fluid’s free surface. The newly developed numerical method has proven its effectiveness in handling complex fluid flow scenarios, including confined flows and extrudate swell simulations of Boger fluids. Furthermore, a new semi-analytical solution for velocity and stress fields is derived, considering fully developed flows of a K–BKZ–PSM fluid in a pipe.

## 1. Introduction

Discovered and developed in the late twentieth century, viscoelastic materials have been used in a number of different applications (polymer industry, biomedicine, automotive industry, food, paints, etc.). Their use is often based on trial and error procedures, resulting in wasting raw material and time before a good end product is achieved. To mitigate this problem, numerical simulations are often used to predict the best material processing conditions. Usually, the simulations are based on the finite element, finite volume and finite difference methods, and the constitutive equations are, in most cases, defined by rheological differential models, such as Oldroyd-B [1], UCM (Upper-convected Maxwell) [2,3], PTT (Phan–Thien–Tanner) [4,5], FENE-P (Finite Extensible Nonlinear Elastic Peterlin) [6,7] and Giesekus models [7]. Simulations of three-dimensional real-world applications require a great deal of computational effort, making the convergence of the algorithms in a reasonable amount of time a difficult task [4]. However, recent technological advances in scientific computing and the development of faster computers have led researchers to perform simulations in more complex geometries and use more sophisticated rheological models (that use integral equations instead of partial differential equations).

It is known that integral constitutive equations can better model various viscoelastic fluids, such as high-density polyethylene (HDPE) [8,9] and low-density polyethylene (LDPE) [10] (used in the injection molding industry), and one of the most successful integral models is the K–BKZ–PSM [11,12,13] (see also [14,15]). Therefore, there is significant interest among research groups worldwide in developing numerical methods to deal with the K–BKZ model, particularly with an emphasis on its application to polymer flows. Many studies have focused on simulating data and phenomena associated with polymer melt flows in rheology and polymer processing; however, there is still a need for further efforts to tackle numerical solutions of the K–BKZ–PSM for three-dimensional, time-dependent, free surface flows.

The vast majority of problems studied in the literature (considering integral models) are about confined flows, such as entry flows [9,16,17] and flows in abrupt contractions [18,19,20,21,22]. Regarding free surface flows, Mitsoulis and Malamataris [20] extended the implementation of the free boundary condition (FBC) method to viscoelastic fluids governed by integral constitutive equations. Specifically, they focused on the K–BKZ–PSM model. To validate their numerical approach, they used the finite element method (FEM) to obtain results in simple test cases, including planar flow at an angle and Poiseuille flow in a tube, where analytical solutions are available for comparison. Furthermore, they have applied the FBC method to the K–BKZ–PSM model using data from a benchmark polymer melt, specifically the IUPAC-LDPE melt. Some other researchers have also considered flows with free surfaces [8,14,23,24,25,26,27]. Ganvir et al. [25] developed a novel approach for simulating extrudate swell using an Arbitrary Lagrangian Eulerian (ALE) technique in conjunction with a finite element formulation. The constitutive behavior of the melt was modeled using a differential exponential Phan–Thien–Tanner (PTT) viscoelastic model. With the proposed method, they have conducted simulations of extrudate swell in both planar and axisymmetric extrusion scenarios, which involve an abrupt contraction ahead of the die exit. Regarding three-dimensional (3D) flows, Rasmussen [28] developed a Galerkin finite element method for simulating three-dimensional transient viscoelastic flows. The method used a Lagrangian kinematic description and integral constitutive models. The numerical implementation was validated with the calculation of various transient and steady drag correction factors for the motion of a sphere in a cylinder containing an upper convected Maxwell fluid. Later, Marín and Rasmussen [29] extended the Galerkin finite element method for simulating three-dimensional transient and non-isothermal flows of K–BKZ type fluids. Tomé et al. [27] proposed a novel numerical approach to tackle the simulation of 3D viscoelastic unsteady free surface flows governed by the Oldroyd-B differential constitutive equation. The numerical method involves solving the governing equations using a finite difference approach on a 3D-staggered grid. To validate the accuracy and reliability of the proposed technique, an exact solution of the flow of an Oldroyd-B fluid inside a 3D-pipe was employed. The results obtained through numerical simulations included the analysis of transient extrudate swell and jet buckling.

Summarizing, previous studies in the field of free surface flows have predominantly centered around two-dimensional (2D) scenarios and used the finite element method. These investigations primarily revolve around the extrudate swell problem, considering both steady and unsteady flows; however, it is worth noting that these studies relied on differential viscoelastic constitutive equations. Therefore, this work introduces a novel numerical method specifically designed to address 3D unsteady free surface flows incorporating integral viscoelastic constitutive equations, specifically, the K–BKZ–PSM model. The key innovation lies in the development of a robust numerical method for integral models using the finite difference method on a staggered grid, which enables accurate predictions of extrudate swell phenomena. We also derive a new semi-analytical solution for the fully developed flow of a K–BKZ–PSM viscoelastic fluid, which can serve for other authors to verify their own numerical implementations of the K–BKZ–PSM integral viscoelastic model.

It is worth noting that the FEM prominently features in the limited body of work concerning this subject. Nevertheless, both FEM and FDM stand as extensively used numerical approaches for tackling partial differential equations (PDEs). When properly employed within suitable conditions, both techniques exhibit stability. Within our research group, a longstanding tradition exists regarding leveraging the finite difference method [2,4,27,30,31,32], resulting in a profound mastery of its implementation. Furthermore, the group has made innovative strides in enhancing the fundamental finite difference methodology. This progression equips us to adeptly handle different grid structures and a range of discretization choices. Consequently, this method takes precedence in our current work.

The paper is structured as follows. In Section 2, we introduce the governing equations for isothermal and incompressible viscoelastic flows modelled by the K–BKZ–PSM constitutive equation. Section 3 is devoted to the numerical method, where the variant of the marker particle method that employs the finite difference method on a staggered grid is described for 3D flows using the K–BKZ–PSM viscoelastic integral model. In Section 4, we derive a new semi-analytical solution for the fully developed flow of a K–BKZ–PSM viscoelastic fluid. For validation of the newly developed numerical method, two case studies are analyzed in Section 5, the confined pipe flow and the extrudate swell free surface flow of a Boger fluid. The paper ends with the conclusions in Section 6.

## 2. Governing Equations

The isothermal and incompressible fluid flow considered in this work is governed by the dimensionless continuity and linear momentum equations [27],

(1) $$\begin{array}{ccc} \nabla \;\cdot \;v & = & 0, \end{array}$$

(2) $$\begin{array}{ccc} \frac{\partial v}{\partial t}+\nabla \;\cdot \;\left(vv\right) & = & -\nabla p+\frac{1}{Re}\nabla^{2}v+\nabla \;\cdot \;\Phi +\frac{1}{Fr^{2}}g, \end{array}$$

together with a constitutive equation for the stress. Φ is a stress tensor given by

(3) $$\Phi =\tau -\frac{1}{Re}\dot{\gamma }\;,\mathrm{with}\dot{\gamma }=\nabla v+{\left(\nabla v\right)}^{T},$$

where τ is a non-Newtonian stress tensor, v(u,v,w) is the velocity field, *p* is the kinematic pressure, g is the gravity acceleration vector and *t* is the time. In these equations, $Fr=U/\sqrt{Lg}$ is the Froude number, $Re=\rho_{0}UL/\eta_{0}$ is the Reynolds number, $\eta_{0}$ is the zero-shear-rate viscosity, $\rho_{0}$ is the fluid density, *g* the magnitude of the gravity acceleration vector and *U* and *L* are the characteristic velocity and length scales, respectively. Note that all variables are dimensionless, with: $x=\bar{x}/L$, $v=\bar{v}/U$, $t=\bar{t}\;U/L$, $p=\bar{p}/\left(\rho U^{2}\right)$ and $\Phi =\bar{\Phi }/\left(\rho U^{2}\right)$.

The constitutive equation for the non-Newtonian stress tensor is given by the K–BKZ–PSM model [11],

(4) $$\tau \left(t\right)=\int_{-\infty }^{t}M\left(t-t^{\prime }\right)H\left(I_{1},I_{2}\right)B_{t^{\prime }}\left(t\right)dt^{\prime }\;,$$

where

(5) $$M\left(t-t^{\prime }\right)=\sum_{k=1}^{m_{1}}\frac{a_{k}}{\lambda_{k}Wi}e^{-\frac{t-t^{\prime }}{\lambda_{k}Wi}},$$

is the memory function, $\lambda_{k}$ is the relaxation time of the fluid, $a_{k}$ is a model parameter and $m_{1}$ is the number of modes. $H(I_{1},I_{2})$ is the Papanastasiou–Scriven–Macosko decay function, being given by

(6) $$H\left(I_{1},I_{2}\right)=\frac{\alpha }{\alpha -3+\beta I_{1}+\left(1-\beta \right)I_{2}}.$$

$B_{t^{\prime }}\left(t\right)$ is the Finger tensor, and $I_{1}=tr\left[B_{t^{\prime }}\left(t\right)\right]$, $I_{2}=\frac{1}{2}\left({\left(I_{1}\right)}^{2}-tr\left[B_{t^{\prime }}^{2}\left(t\right)\right]\right)$ are the first and second invariants of $B_{t^{\prime }}\left(t\right)$, respectively. The parameters $a_{k},\;\lambda_{k},\;\alpha$ and β are obtained from a fit to rheological data. $Wi=\lambda_{ref}U/L$ is the Weissenberg number, the viscosity is given by $\eta_{0}=\sum_{k=1}^{m_{1}}a_{k}\lambda_{k}$ and $\lambda_{ref}=\sum_{k=1}^{m_{1}}\frac{a_{k}\lambda_{k}^{2}}{a_{k}\lambda_{k}}$ is the mean relaxation time [14].

In this work, the method of *deformation fields* [33] is used to update the Finger tensor as the fluid flows. In this methodology, (N+1)-deformation instants ($t^{\prime }$) are defined in the interval [0,t] where the history of deformation is stored. This deformation is updated by solving the transport equation for $B_{t^{\prime }}\left(t\right)$,

(7) $$\begin{array}{cc} \frac{\partial }{\partial t}B_{t^{\prime }}\left(x,t\right)+v\left(x,t\right)\;\cdot \;\nabla B_{t^{\prime }}\left(x,t\right)= & {\left[\nabla v(x,t)\right]}^{T}\;\cdot \;B_{t^{\prime }}\left(x,t\right)+B_{t^{\prime }}\left(x,t\right)\;\cdot \;\nabla v\left(x,t\right)\;. \end{array}$$

The governing equations are solved in a Cartesian 3D system (x,y,z,t) where

p=p(x,y,z,t),

$$v={\left(u\left(x,y,z,t\right),\;v\left(x,y,z,t\right),\;w\left(x,y,z,t\right)\right)}^{T},$$

$$\tau \left(x,y,z,t\right)=\left[\begin{array}{ccc} \tau^{xx} & \tau^{xy} & \tau^{xz}\\ \tau^{xy} & \tau^{yy} & \tau^{yz}\\ \tau^{xz} & \tau^{yz} & \tau^{zz} \end{array}\right]\;\mathrm{and}\;B_{t^{\prime }\left(t\right)}\left(x,y,z,t\right)=\left[\begin{array}{ccc} B^{xx} & B^{xy} & B^{xz}\\ B^{xy} & B^{yy} & B^{yz}\\ B^{xz} & B^{yz} & B^{zz} \end{array}\right].$$

This results in the following system of equations that need to be solved for the pressure, velocity and stress:

*continuity equation*:

(8) $$\frac{\partial u}{\partial x}+\frac{\partial v}{\partial y}+\frac{\partial w}{\partial z}=0.$$

*linear momentum equations*:

(9) $$\begin{array}{cc} \frac{\partial u}{\partial t}+\frac{\partial (uu)}{\partial x}+\frac{\partial (vu)}{\partial y}+\frac{\partial (wu)}{\partial z}= & -\frac{\partial p}{\partial x}+\frac{1}{Re}\left[\frac{\partial^{2}u}{\partial x^{2}}+\frac{\partial^{2}u}{\partial y^{2}}+\frac{\partial^{2}u}{\partial z^{2}}\right]\\ & +\frac{\partial \Phi^{xx}}{\partial x}+\frac{\partial \Phi^{xy}}{\partial y}+\frac{\partial \Phi^{xz}}{\partial z}+\frac{1}{Fr^{2}}g_{x},\\ \frac{\partial v}{\partial t}+\frac{\partial (uv)}{\partial x}+\frac{\partial (vv)}{\partial y}+\frac{\partial (wv)}{\partial z}= & -\frac{\partial p}{\partial y}+\frac{1}{Re}\left[\frac{\partial^{2}v}{\partial x^{2}}+\frac{\partial^{2}v}{\partial y^{2}}+\frac{\partial^{2}v}{\partial z^{2}}\right]\\ & +\frac{\partial \Phi^{xy}}{\partial x}+\frac{\partial \Phi^{yy}}{\partial y}+\frac{\partial \Phi^{yz}}{\partial z}+\frac{1}{Fr^{2}}g_{y},\\ \frac{\partial w}{\partial t}+\frac{\partial (uw)}{\partial x}+\frac{\partial (vw)}{\partial y}+\frac{\partial (ww)}{\partial z}= & -\frac{\partial p}{\partial z}+\frac{1}{Re}\left[\frac{\partial^{2}w}{\partial x^{2}}+\frac{\partial^{2}w}{\partial y^{2}}+\frac{\partial^{2}w}{\partial z^{2}}\right]\\ & +\frac{\partial \Phi^{xz}}{\partial x}+\frac{\partial \Phi^{yz}}{\partial y}+\frac{\partial \Phi^{zz}}{\partial z}+\frac{1}{Fr^{2}}g_{z}, \end{array}$$

where $g_{x},g_{y},g_{z}$ are the Cartesian components of the gravity vector.

*stress tensor* Φ:

(10) $$\begin{array}{cc} \Phi^{xx} & =\tau^{xx}-\frac{2}{Re}\frac{\partial u}{\partial x},\\ \Phi^{xy} & =\tau^{xy}-\frac{1}{Re}\left[\frac{\partial u}{\partial y}+\frac{\partial v}{\partial x}\right],\\ \Phi^{xz} & =\tau^{xz}-\frac{1}{Re}\left[\frac{\partial u}{\partial z}+\frac{\partial w}{\partial x}\right],\\ \Phi^{yy} & =\tau^{yy}-\frac{2}{Re}\frac{\partial v}{\partial y},\\ \Phi^{yz} & =\tau^{yz}-\frac{1}{Re}\left[\frac{\partial v}{\partial z}+\frac{\partial w}{\partial y}\right],\\ \Phi^{zz} & =\tau^{zz}-\frac{2}{Re}\frac{\partial w}{\partial z}. \end{array}$$

*stress tensor **τ***:

(11) $$\begin{array}{cc} \tau^{xx}\left(t\right) & =\int_{-\infty }^{t}\sum_{k=1}^{m_{1}}\frac{a_{k}}{Wi\lambda_{k}}e^{-\frac{\left(t-t^{\prime }\right)}{Wi\lambda_{k}}}\frac{\alpha }{\alpha -3+\beta I_{1}+\left(1-\beta \right)I_{2}}B_{t^{\prime }}^{xx}\left(t\right)dt^{\prime },\\ \tau^{xy}\left(t\right) & =\int_{-\infty }^{t}\sum_{k=1}^{m_{1}}\frac{a_{k}}{Wi\lambda_{k}}e^{-\frac{\left(t-t^{\prime }\right)}{Wi\lambda_{k}}}\frac{\alpha }{\alpha -3+\beta I_{1}+\left(1-\beta \right)I_{2}}B_{t^{\prime }}^{xy}\left(t\right)dt^{\prime },\\ \tau^{xz}\left(t\right) & =\int_{-\infty }^{t}\sum_{k=1}^{m_{1}}\frac{a_{k}}{Wi\lambda_{k}}e^{-\frac{\left(t-t^{\prime }\right)}{Wi\lambda_{k}}}\frac{\alpha }{\alpha -3+\beta I_{1}+\left(1-\beta \right)I_{2}}B_{t^{\prime }}^{xz}\left(t\right)dt^{\prime },\\ \tau^{yy}\left(t\right) & =\int_{-\infty }^{t}\sum_{k=1}^{m_{1}}\frac{a_{k}}{Wi\lambda_{k}}e^{-\frac{\left(t-t^{\prime }\right)}{Wi\lambda_{k}}}\frac{\alpha }{\alpha -3+\beta I_{1}+\left(1-\beta \right)I_{2}}B_{t^{\prime }}^{yy}\left(t\right)dt^{\prime },\\ \tau^{yz}\left(t\right) & =\int_{-\infty }^{t}\sum_{k=1}^{m_{1}}\frac{a_{k}}{Wi\lambda_{k}}e^{-\frac{\left(t-t^{\prime }\right)}{Wi\lambda_{k}}}\frac{\alpha }{\alpha -3+\beta I_{1}+\left(1-\beta \right)I_{2}}B_{t^{\prime }}^{yz}\left(t\right)dt^{\prime },\\ \tau^{zz}\left(t\right) & =\int_{-\infty }^{t}\sum_{k=1}^{m_{1}}\frac{a_{k}}{Wi\lambda_{k}}e^{-\frac{\left(t-t^{\prime }\right)}{Wi\lambda_{k}}}\frac{\alpha }{\alpha -3+\beta I_{1}+\left(1-\beta \right)I_{2}}B_{t^{\prime }}^{zz}\left(t\right)dt^{\prime }. \end{array}$$

*Finger tensor* B:

(12) $$\begin{array}{cc} \frac{\partial B^{xx}}{\partial t}+\frac{\partial (uB^{xx})}{\partial x}+\frac{\partial (vB^{xx})}{\partial y}+\frac{\partial (wB^{xx})}{\partial z}= & 2\left[\frac{\partial u}{\partial x}B^{xx}+\frac{\partial u}{\partial y}B^{xy}+\frac{\partial u}{\partial z}B^{xz}\right],\\ \frac{\partial B^{xy}}{\partial t}+\frac{\partial (uB^{xy})}{\partial x}+\frac{\partial (vB^{xy})}{\partial y}+\frac{\partial (wB^{xy})}{\partial z}= & \frac{\partial v}{\partial x}B^{xx}+\left[\frac{\partial u}{\partial x}+\frac{\partial v}{\partial y}\right]B^{xy}\\ & +\frac{\partial v}{\partial z}B^{xz}+\frac{\partial u}{\partial y}B^{yy}+\frac{\partial u}{\partial z}B^{yz},\\ \frac{\partial B^{xz}}{\partial t}+\frac{\partial (uB^{xz})}{\partial x}+\frac{\partial (vB^{xz})}{\partial y}+\frac{\partial (wB^{xz})}{\partial z}= & \frac{\partial w}{\partial x}B^{xx}+\frac{\partial w}{\partial y}B^{xy}+\left[\frac{\partial u}{\partial x}+\frac{\partial w}{\partial z}\right]B^{xz}\\ & +\frac{\partial u}{\partial y}B^{yz}+\frac{\partial u}{\partial z}B^{zz},\\ \frac{\partial B^{yy}}{\partial t}+\frac{\partial (uB^{yy})}{\partial x}+\frac{\partial (vB^{yy})}{\partial y}+\frac{\partial (wB^{yy})}{\partial z}= & 2\left[\frac{\partial v}{\partial x}B^{xy}+\frac{\partial v}{\partial y}B^{yy}+\frac{\partial v}{\partial z}B^{yz}\right],\\ \frac{\partial B^{yz}}{\partial t}+\frac{\partial (uB^{yz})}{\partial x}+\frac{\partial (vB^{yz})}{\partial y}+\frac{\partial (wB^{yz})}{\partial z}= & \frac{\partial w}{\partial x}B^{xy}+\frac{\partial v}{\partial x}B^{xz}+\frac{\partial w}{\partial y}B^{yy}\\ & +\left[\frac{\partial v}{\partial y}+\frac{\partial w}{\partial z}\right]B^{yz}+\frac{\partial v}{\partial z}B^{zz},\\ \frac{\partial B^{zz}}{\partial t}+\frac{\partial (uB^{zz})}{\partial x}+\frac{\partial (vB^{zz})}{\partial y}+\frac{\partial (wB^{zz})}{\partial z}= & 2\left[\frac{\partial w}{\partial x}B^{xz}+\frac{\partial w}{\partial y}B^{yz}+\frac{\partial w}{\partial z}B^{zz}\right]. \end{array}$$

## 3. Numerical Method

The governing equations are solved by a variant of the marker particle method [4,27], which employs the finite difference method on a staggered grid. This methodology is implemented in the FREEFLOW-3D code developed by researchers from the Institute of Mathematical and Computing Sciences (ICMC) at the University of São Paulo (USP) in Brazil. Code details can be found in [4,27,30,31] considering 2D, 3D and radial symmetry flows. The precision of the numerical technique and its validation for three-dimensional viscoelastic flows with a free surface is presented in the works of Tomé et al. [27,30] (which only uses differential constitutive equations). The use of integral models in three-dimensional flows (considering free surface problems) has not yet been tested on this system (see Tomé et al. [4,27,30,31]). The novelty of this work is to incorporate equations for viscoelastic fluids using integral models (more complex than the differential type models) in the FREEFLOW-3D system.

**Figure 1:**
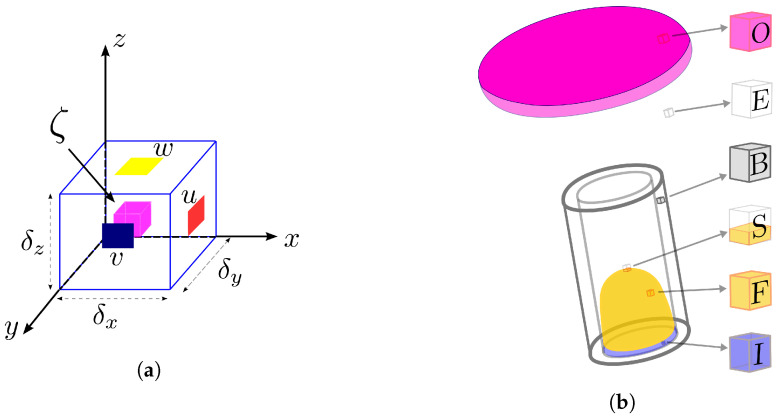
(**a**) Typical three-dimensional staggered cell and (**b**) illustration of cell type classification used.

In this methodology, the velocity field is approximated in the face of the cells, and the other variables, denoted by ζ, are evaluated in the center of the computational cells (see Figure 1a). The technique adopted here is presented by Tomé et al. [30,31] for differential models, where the free surface is defined by marker particles that move with the local fluid velocity. In addition, the computational cells are defined as (see also Figure 1b):

*■*  Fluid entrance: *Inflow — I*,
*■*  Fluid exit: *Outflow — O*,
*■*  Rigid boundaries: *Boundary — B*,
*■*  Empty cells: *Empty — E*,
*■*  Free surface cells: *Surface — S*,
*■*  Full cells: *Full — F*.

To solve Equations (1) and (2), one must specify boundary conditions for the velocity field. A velocity field ($V_{inf}$) is prescribed in the fluid inlet cells (*inflows*), and a fully developed flow is assumed in the *outflow* (a homogeneous Neumann boundary condition ∂v/∂n=0 is assumed, where *n* is the normal direction to the contour). In the fluid inlet cells (*inflows*), we assume that $\frac{\partial p}{\partial n}=0$ and the Finger tensor $B_{t^{\prime }}$ is the identity matrix. In outflow regions, we assume Neumann conditions for Finger tensor $\left(\frac{\partial B}{\partial n}=0\right)$, and we assume p=0. We also take v=0 in rigid boundaries. Details of the boundary conditions adopted in this work can be found in Tomé et al. [30] or Castelo et al. [31].

The solutions $v\left(x,t_{n+1}\right),\;p\left(x,t_{n+1}\right)$ and $\tau \left(x,t_{n+1}\right)$ at time step $t_{n+1}=t+\Delta t$ are obtained in the following way: first, using the values of $\tau (x,t_{n})$, the velocity and pressure fields at time $t_{n+1}$ are calculated. Then, $v\left(x,t_{n+1}\right)$ is used to calculate the tensor $\tau \left(x,t_{n+1}\right)$ by the method of deformation fields, and, lastly, the free surface is updated. Specifically, the following steps are performed:

**Step 1** — *Calculation of $v\left(x,t_{n+1}\right)$ and $p\left(x,t_{n+1}\right)$*

It is assumed that, at time *t*, the variables $v\left(x,t\right)=v^{\left(n\right)},p\left(x,t\right)=p^{\left(n\right)},\tau \left(x,t\right)=\tau^{\left(n\right)}$ and the marker’s positions $x\left(t\right)=x^{\left(n\right)}$ are known. Then, $v\left(x,t_{n+1}\right)$ and $p\left(x,t_{n+1}\right)$ are obtained as follows:

1. Calculate ${\dot{\gamma }}^{\left(n\right)}=\left[\nabla v^{\left(n\right)}+{\left(\nabla v^{\left(n\right)}\right)}^{T}\right]$, and, from the EVSS [34] transformation, obtain $\Phi =\tau^{\left(n\right)}-\frac{1}{Re}{\dot{\gamma }}^{\left(n\right)}$;

2. Calculate an intermediate velocity field ${\tilde{v}}^{\left(n+1\right)}$ using the ideas of the projection method [30,31] to uncouple the conservation of mass and momentum equations. An intermediate velocity field ${\tilde{v}}^{\left(n+1\right)}$ is obtained from Equation (2) using explicit Euler Methods, where $p^{\left(n\right)}$ is an approximation to $p^{\left(n+1\right)}$. The boundary conditions for ${\tilde{v}}^{\left(n+1\right)}$ are the same as those for the final velocity $v^{\left(n+1\right)}$. Details of boundary conditions for full cells (F), outflow cells (O) and free surface cells (S) are provided in detail in Tomé et al. [4,30] and will not be presented here for the sake of conciseness. It can be shown that ${\tilde{v}}^{\left(n+1\right)}$ possesses the correct vorticity at time $t_{n+1}$, but it does not conserve mass in general. Therefore, there is a potential function $\psi^{\left(n+1\right)}$ so that,

(13) $$v^{\left(n+1\right)}={\tilde{v}}^{\left(n+1\right)}-\nabla \psi^{\left(n+1\right)}.$$

3. Solve the Poisson equation for the potential function ψ for every F-cell in the domain,

(14) $$\nabla^{2}\psi^{\left(n+1\right)}=\nabla \;\cdot \;{\tilde{v}}^{\left(n+1\right)}.$$

The boundary conditions required for solving this Poisson equation are the homogeneous Neumann conditions for rigid walls and inflows, while homogeneous Dirichlet conditions are used at outflows.

4. Compute the final velocity field from Equation (13);

5. Compute the final pressure field (see [4]) by

(15) $$p^{\left(n+1\right)}=p^{\left(n\right)}+\frac{\psi^{\left(n+1\right)}}{\Delta t}.$$

Details of the discretization of the equations (temporal and spatial) considering all types of cells (see Figure 1b) are given in [4,27,30].

**Step 2** — *Calculation of the extra stress tensor $\tau \left(x,t_{n+1}\right)$ and free surface update*

To calculate the extra stress tensor $\tau \left(x,t_{n+1}\right)$, initially, the Finger tensor is updated at $t_{n+1}$ for every full cell (F) and surface cell (S) for every intermediate time $t^{\prime }$ using Equation (7). Details of the calculation of the Finger B tensor (in two dimensions) can be found in [32] and will not be presented here because the extension to three dimensions is straightforward. Note that, for each computational cell (F and S), Equation (7) is solved N times (for each $t^{\prime }$), considering each of the components of the Finger tensor. Thus, considering three-dimensional flows, the computational cost to obtain the deformation history in each cell is high, demanding a great deal of memory and simulation time (since, for each cell, it is necessary to calculate the Finger tensor N times for each component of the deformation matrix). For inflow cells (I), boundary cells (B) and empty cells (E), the Finger tensor is the identity tensor. In the outflow, the Neumann condition is assumed.

The definition of the points $t^{\prime }$ for the calculation of the components of the Finger tensor and the tensor τ are given as follows. Let $t_{j}^{\prime },\;j=0,1,\cdots ,N$, be (N+1)-points in the interval $\left[0,t_{n+1}\right]$. Then, the constitutive equation can be written in the form

(16) $$\begin{array}{cc} \tau \left(t_{n+1}\right)= & \int_{-\infty }^{0}M\left(t_{n+1}-t^{\prime }\right)H\left(I_{1},I_{2}\right)B_{t^{\prime }}\left(t_{n+1}\right)dt^{\prime }\\ \; & +\sum_{j=0}^{\frac{N-2}{2}}\int_{t_{2j}^{\prime }}^{t_{2j+2}^{\prime }}M\left(t_{n+1}-t^{\prime }\right)H\left(I_{1},I_{2}\right)B_{t^{\prime }}\left(t_{n+1}\right)dt^{\prime }, \end{array}$$

where an even *N* is assumed. For $t^{\prime }<0,\;B_{t^{\prime }}\left(t_{n+1}\right)=B_{0}\left(t_{n+1}\right)$, and, therefore, the first integral becomes

(17) $$\int_{-\infty }^{0}M\left(t_{n+1}\right)H\left(I_{1}\left(B_{0}\left(t_{n+1}\right)\right),I_{2}\left(B_{0}\left(t_{n+1}\right)\right)\right)B_{0}\left(t_{n+1}\right)dt^{\prime },$$

and can be solved exactly.

Each integral under the summation operator $\int_{t_{2j}^{\prime }}^{t_{2j+2}^{\prime }}M\left(t_{n+1}-t^{\prime }\right)H\left(I_{1},I_{2}\right)B_{t^{\prime }}\left(t_{n+1}\right)dt^{\prime }$ is approximated by the 3-points quadrature formula

(18) $$\begin{array}{cc} I_{3}= & A_{0}\times H\left(I_{1}\left(B_{t_{2j}^{\prime }}\left(t_{n+1}\right)\right),I_{2}\left(B_{t_{2j}^{\prime }}\left(t_{n+1}\right)\right)\right)B_{t_{2j}^{\prime }}\left(t_{n+1}\right)\\ \; & +A_{1}\times H\left(I_{1}\left(B_{t_{2j+1}^{\prime }}\left(t_{n+1}\right)\right),I_{2}\left(B_{t_{2j+1}^{\prime }}\left(t_{n+1}\right)\right)\right)B_{t_{2j+1}^{\prime }}\left(t_{n+1}\right)\\ \; & +A_{2}\times H\left(I_{1}\left(B_{t_{2j+2}^{\prime }}\left(t_{n+1}\right)\right),I_{2}\left(B_{t_{2j+2}^{\prime }}\left(t_{n+1}\right)\right)\right)B_{t_{2j+2}^{\prime }}\left(t_{n+1}\right). \end{array}$$

The coefficients $A_{0},A_{1},A_{2}$ are obtained by solving the (3×3) linear system

(19) $$\begin{array}{cc} A_{0}+A_{1}+A_{2} & =b_{0}=\int_{t_{2j}^{\prime }}^{t_{2j+2}^{\prime }}M\left(t_{n+1}-t^{\prime }\right)dt^{\prime },\\ A_{0}\times t_{2j}^{\prime }+A_{1}\times t_{2j+1}^{\prime }+A_{2}\times t_{2j+2}^{\prime } & =b_{1}=\int_{t_{2j}^{\prime }}^{t_{2j+2}^{\prime }}M\left(t_{n+1}-t^{\prime }\right)t^{\prime }dt^{\prime },\\ A_{0}\times {\left(t_{2j}^{\prime }\right)}^{2}+A_{1}\times {\left(t_{2j+1}^{\prime }\right)}^{2}+A_{2}\times {\left(t_{2j+2}^{\prime }\right)}^{2} & =b_{2}=\int_{t_{2j}^{\prime }}^{t_{2j+2}^{\prime }}M\left(t_{n+1}-t^{\prime }\right){\left(t^{\prime }\right)}^{2}dt^{\prime }, \end{array}$$

and are found to be

(20) $$\begin{array}{cc} \; & A_{2}=\frac{t_{2j+1}^{\prime }t_{2j}^{\prime }b_{0}+b_{2}-t_{2j}^{\prime }b_{1}-t_{2j+1}^{\prime }b_{1}}{{\left(t_{2j+2}^{\prime }\right)}^{2}-t_{2j+1}^{\prime }t_{2j+2}^{\prime }+t_{2j+1}^{\prime }t_{2j}^{\prime }-t_{2j}^{\prime }t_{2j+2}^{\prime }},\\ \; & A_{1}=\frac{-t_{2j}^{\prime }b_{1}+t_{2j}^{\prime }b_{0}t_{2j+2}^{\prime }+b_{2}-t_{2j+2}^{\prime }b_{1}}{\left(t_{2j+1}^{\prime }-t_{2j}^{\prime }\right)\left(t_{2j+1}^{\prime }-t_{2j+2}^{\prime }\right)},\\ \; & A_{0}=-\frac{-t_{2j+1}^{\prime }b_{1}+t_{2j+1}^{\prime }b_{0}t_{2j+2}^{\prime }+b_{2}-t_{2j+2}^{\prime }b_{1}}{\left(-t_{2j+2}^{\prime }+t_{2j}^{\prime }\right)\left(t_{2j+1}^{\prime }-t_{2j}^{\prime }\right)}. \end{array}$$

One of the key issues of the deformation fields method is how the integration nodes $0=t_{0}^{\prime }<$$t_{1}^{\prime }<\cdots <t_{N}^{\prime }=t_{n+1}$ are distributed over the interval $\left[0,t_{n+1}\right]$ because such distribution can affect the accuracy of the results when solving complex flows. In this work, we used an ad hoc methodology for the discretization of the interval $\left[0,t_{n+1}\right]$, where a geometric progression is employed to calculate the integration nodes. Note that we consider time-dependent flows, and, therefore, the integration nodes are calculated at time $t_{n+1}$ as follows:

(a) Set $t_{0}^{\prime }=0$ and $t_{N}^{\prime }=t_{n+1}$;
(b) $t_{N-j}^{\prime }=t_{N}^{\prime }-q^{j},j=1,2,\cdots ,N-1$, where $q={\left(t_{n+1}/\Delta t\right)}^{1/N}$.

The last step in the calculation is to update the position of the moving free surface (the S-cell in Figure 1b). The fluid surface is represented by a piecewise linear surface composed of triangles and quadrilaterals having marker particles on their vertices (see [30]). The particle coordinates, stored at each time step, are updated, solving the equation

(21) $$\frac{dx}{dt}=v,$$

by Euler’s method. With the new coordinates of each marker particle, a reclassification of the free surface cells is performed. A free surface cell can become an empty cell (E-cell in Figure 1b) or a full cell (F-cell in Figure 1b) or remain an S-type cell. Details on the marker particles that define the free surface and the steps for inserting and removing particles will not be shown here, but the reader can consult Tomé et al. [30] or Castelo et al. [31].

## 4. Semi-Analytical Solution

**Figure 2:**
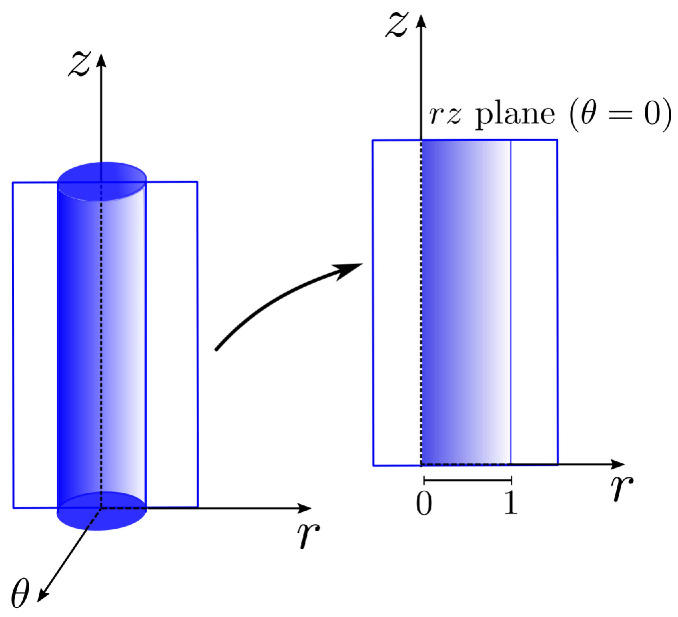
Representation of the pipe and a section in the rz plane.

We will now derive a semi-analytical solution for a fully developed three-dimensional tube flow of a K–BKZ–PSM fluid to validate the numerical implementation. Due to the complexity of the integral model, some simplifications need to be assumed to develop the analytical solution, which is only possible for some types of domains. We consider cylindrical coordinates (see Figure 2) for simplicity and assume pure shear flow. After finding the semi-analytical solution, we present the change in variables to obtain the solution in Cartesian coordinates.

We assume that $r\;\in \;\left[0,\;1\right],u=0,w^{in}=w\left(r\right),\dot{\gamma }=\frac{\partial w}{\partial r},$ and

(22) $$B\left(r,\theta ,z\right)=\left[\begin{array}{ccc} 1 & 0 & \dot{\gamma }\left(t-t^{\prime }\right)\\ 0 & 1 & 0\\ \dot{\gamma }\left(t-t^{\prime }\right) & 0 & 1+{\dot{\gamma }}^{2}{\left(t-t^{\prime }\right)}^{2} \end{array}\right].$$

The invariants $I_{1}$ and $I_{2}$ that are required in the Papanastasiou function $H(I_{1},I_{2})$ take the form

(23) $$I_{1}=I_{2}=3+{\dot{\gamma }}^{2}{\left(t-t^{\prime }\right)}^{2},$$

and the tensor components are given by

(24) $$\begin{array}{cc} \tau^{rr}=\tau^{\theta \theta } & =\frac{a_{1}\alpha }{Wi}\int_{-\infty }^{t}\frac{e^{-(t-t^{\prime })/Wi}}{\alpha +{\dot{\gamma }}^{2}{\left(t-t^{\prime }\right)}^{2}}dt^{\prime }\;,\\ \tau^{rz} & =\frac{a_{1}\alpha }{Wi}\int_{-\infty }^{t}\frac{\dot{\gamma }\left(t-t^{\prime }\right)e^{-(t-t^{\prime })/Wi}}{\alpha +{\dot{\gamma }}^{2}{\left(t-t^{\prime }\right)}^{2}}dt^{\prime }\;,\\ \tau^{zz} & =\frac{a_{1}\alpha }{Wi}\int_{-\infty }^{t}\frac{\left[1+{\dot{\gamma }}^{2}{\left(t-t^{\prime }\right)}^{2}\right]e^{-(t-t^{\prime })/Wi}}{\alpha +{\dot{\gamma }}^{2}{\left(t-t^{\prime }\right)}^{2}}dt^{\prime }\;. \end{array}$$

Taking the change in variables $s=t-t^{\prime }$, these equations are rewritten as

(25) $$\begin{array}{cc} \tau^{rr}=\tau^{\theta \theta } & =\frac{a_{1}\alpha }{Wi}\int_{0}^{\infty }\frac{1\;e^{-s/Wi}}{\alpha +{\dot{\gamma }}^{2}s^{2}}ds\;,\\ \tau^{rz} & =\frac{a_{1}\alpha }{Wi}\int_{0}^{\infty }\frac{\dot{\gamma }se^{-s/Wi}}{\alpha +{\dot{\gamma }}^{2}s^{2}}ds\;,\\ \tau^{zz} & =\frac{a_{1}\alpha }{Wi}\int_{0}^{\infty }\frac{\left[1+{\dot{\gamma }}^{2}s^{2}\right]e^{-s/Wi}}{\alpha +{\dot{\gamma }}^{2}s^{2}}ds. \end{array}$$

Thus, the equations of continuity and balance of momentum become

(26) $$-\frac{\partial p}{\partial r}+\frac{1}{r}\frac{\partial }{\partial r}\left(r\tau^{rr}\right)-\frac{\tau^{\theta \theta }}{r}=0\;,$$

(27) $$-\frac{\partial p}{\partial z}+\frac{1}{r}\frac{\partial }{\partial r}\left(r\tau^{rz}\right)=0.$$

Integrating Equation (26), we obtain

(28) $$p\left(r,z\right)=\int \frac{1}{r}\frac{\partial }{\partial r}\left(r\tau^{rr}\right)dr-\int \frac{\tau^{\theta \theta }}{r}dr+F\left(z\right).$$

Thus,

(29) $$\frac{\partial p}{\partial z}=F^{\prime }\left(z\right),$$

and Equation (27) is rewritten as

(30) $$\frac{1}{r}\frac{\partial }{\partial r}\left(r\tau^{rz}\right)=F^{\prime }\left(z\right).$$

The left hand side of Equation (30) is just a function of *r*, so $F^{\prime }$ must be constant, let us say C, and therefore C=dp/dz. In this way, it follows that

(31) $$\tau^{rz}\left(r\right)=\frac{1}{2}Cr+\frac{h(z)}{r},$$

where $\tau^{rz}\left(r=0\right)$ should be finite and therefore h(z)=0, leading to

(32) $$\tau^{rz}\left(r\right)=\frac{1}{2}Cr.$$

The second equation in Equation (25) can then be rewritten as

(33) $$\frac{1}{2}Cr=\frac{a_{1}\alpha }{Wi}\int_{0}^{\infty }\frac{\dot{\gamma }se^{-s/Wi}}{\alpha +{\dot{\gamma }}^{2}s^{2}}ds.$$

The inlet boundary condition allows one to determine the constant *C*. The inflow velocity $w^{in}\left(r\right)$ is given by

(34) $$w^{in}\left(r\right)=1-r^{2},\;\;\;where\;\;w^{in}\left(0\right)=1\;and\;w^{in}\left(1\right)=0,$$

thus,

(35) $$\int_{0}^{1}rw^{in}dr=\int_{0}^{1}r\left(1-r^{2}\right)dr=\frac{1}{4}.$$

By mass conservation,

(36) $$\int_{0}^{1}r\;w^{in}\left(r\right)dr=\frac{1}{4},$$

and integrating by parts leads to

(37) $$\int_{0}^{1}r\;w^{in}\left(r\right)dr={\left[\frac{1}{2}r^{2}w^{in}\left(r\right)\right]}_{0}^{1}-\frac{1}{2}\int_{0}^{1}r^{2}\dot{\gamma }dr,$$

with

(38) $$\int_{0}^{1}r^{2}\dot{\gamma }dr=-\frac{1}{2}.$$

Thus, to determine *C*, we must obtain $\dot{\gamma }\left(r\right)$ from Equation (33) and verify that

(39) $$F\left(C\right)=\int_{0}^{1}r^{2}\dot{\gamma }dr+\frac{1}{2}=0\;issatisfied.$$

The steps to calculate semi-analytical solutions are as follows:

**Step 1:**  Set an interval $\left[C_{0},\;C_{1}\right]$ such that $F\left(C_{0}\right)\times F\left(C_{1}\right)<0$.
**Step 2:**  Determine the zero for |F(C)| taking |F(C)|<ϵ, where ϵ is a small value (ϵ is the tolerance for the error). We carefully selected the value of ϵ to ensure the attainment of a semi-analytical solution accurate to six significant digits. Using Gauss–Laguerre quadrature in Equation (33), obtain $\dot{\gamma }\left(r\right)$. Using Equation (39), obtain the value of F(C) using Simpson 1/3 quadrature.
**Step 3:**  Lastly, determine $\tau^{rr}\left(r\right)$, $\tau^{zz}\left(r\right)$ and $\tau^{rz}\left(r\right)$ using the first and third equations in Equations (25) and (32), respectively.

The solution in three dimensions is obtained by making the change in coordinates as follows:

$$\left(\begin{array}{ccc} \tau^{xx} & \tau^{xy} & \tau^{xz}\\ \tau^{xy} & \tau^{yy} & \tau^{yz}\\ \tau^{xz} & \tau^{yz} & \tau^{zz} \end{array}\right)=XYX^{T}$$

where $X=\left(\begin{array}{ccc} \cos(\theta ) & -\sin(\theta ) & 0\\ \sin(\theta ) & \cos(\theta ) & 0\\ 0 & 0 & 1 \end{array}\right),\;Y=\left(\begin{array}{ccc} \tau^{rr} & 0 & \tau^{rz}\\ 0 & \tau^{\theta \theta } & 0\\ \tau^{rz} & 0 & \tau^{zz} \end{array}\right)\;\mathrm{and}$

(40) $$\begin{array}{cc} \tau^{xx} & =\left[\cos{\left(\theta \right)}^{2}+\sin{\left(\theta \right)}^{2}\right]\tau^{rr},\\ \tau^{xy} & =\left[\cos\left(\theta \right)\sin\left(\theta \right)\right]\tau^{rr}-\left[\cos\left(\theta \right)\sin\left(\theta \right)\right]\tau^{rr},\\ \tau^{xz} & =\cos\left(\theta \right)\tau^{rz},\\ \tau^{yy} & =\left[\cos{\left(\theta \right)}^{2}+\sin{\left(\theta \right)}^{2}\right]\tau^{rr},\\ \tau^{yz} & =\sin\left(\theta \right)\tau^{rz},\\ \tau^{zz} & =\tau^{zz}. \end{array}$$

Thus, in three-dimensional Cartesian coordinates, we have that

(41) $$\begin{array}{cc} \tau^{xx} & =\tau^{rr},\\ \tau^{xy} & =0,\\ \tau^{xz} & =\frac{x}{\sqrt{x^{2}+y^{2}}}\tau^{rz},\\ \tau^{yy} & =\tau^{rr},\\ \tau^{yz} & =\frac{y}{\sqrt{x^{2}+y^{2}}}\tau^{rz},\\ \tau^{zz} & =\tau^{zz}. \end{array}$$

## 5. Results

The numerical code will now be used to solve confined (see Section 5.1) and free surface flows (see Section 5.2).

### 5.1. Confined Pipe Flows

In this confined pipe flow, the fluid is assumed to have only one relaxation mode. Therefore, it is possible to compare the simulation results with the semi-analytical solution presented before. The parameters used in this simulation are (see Tomé et al. [32] and Quinzani et al. [35]):

- Diameter $L=0.01\;m,\;U=0.025\;{m.s}^{-1},\;\rho =801.5\;{\mathrm{Kg}.m}^{-3}$;
- $\lambda_{ref}=0.1396\;s,\;a_{1}=1.6648\;\mathrm{Pa},\;\eta_{0}=0.2324\;\mathrm{Pa}.s$;
- Number of deformation fields N=50;
- $Re=\frac{\rho LU}{\eta_{0}}=0.8621,\;Wi=\lambda_{ref}\frac{U}{L}=0.349$;
- Geometry: 0.01m × 0.01m × 0.05m;
- Meshes (number of cells in the *x*, *y* and *z* directions): M1=12×12×60 ($\delta x=\delta y=\frac{0.01}{12}$), M2=16×16×80 ($\delta x=\delta y=\frac{0.01}{16}$), M3=20×20×100 ($\delta x=\delta y=\frac{0.01}{20}$), M4=24×24×120 ($\delta x=\delta y=\frac{0.01}{24}$) and M5=28×28×140 ($\delta x=\delta y=\frac{0.01}{28}$).

**Figure 3:**
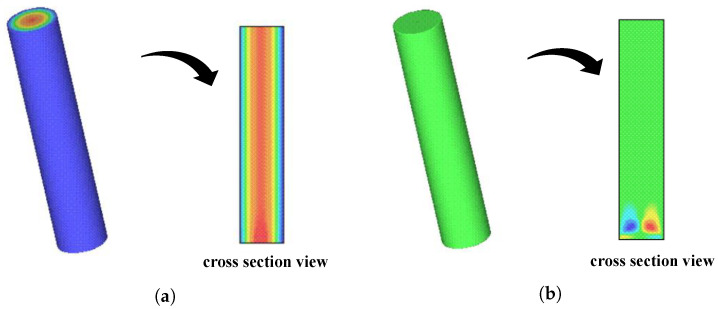
Velocity components *u* and *w* of v(u,v,w) along the plane xz (y=0) at $\bar{t}=20\;s$. (**a**) Visualization of the velocity profile *w*. (**b**) Visualization of the velocity profile *u*.

Figure 3a,b show the velocity profiles *w* and *u*, respectively, with corresponding cross-section velocity distributions in the plane xz (obtained mesh M3). The velocity profiles are fully developed at $\bar{t}=20\;s$, suggesting that they have reached a steady-state condition. The velocity profile *w* shows a parabolic shape in the cross-section represented in Figure 3a, while the influence of the inflow can be observed in the cross-section depicted in Figure 3b, but the solution for *u* exhibits the expected physical behavior outside this region. Notice that, in all full cells, the initial velocity vector is defined as v=(u,v,w)=(0,0,0).

**Figure 4:**
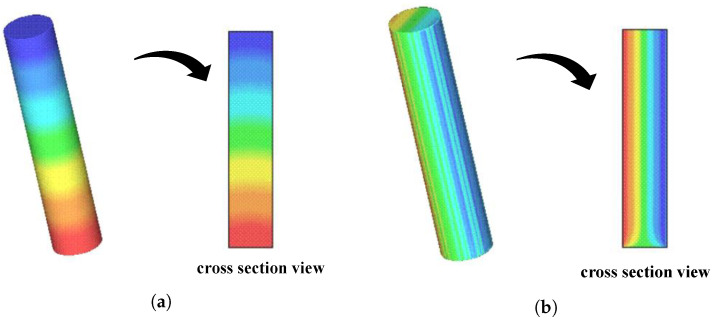
Pressure and $\tau^{xz}$ distribution along the xz plane (y=0). (**a**) Visualization of the pressure *p*. (**b**) Visualization of the $\tau^{xz}$ tensor component.

**Figure 5:**
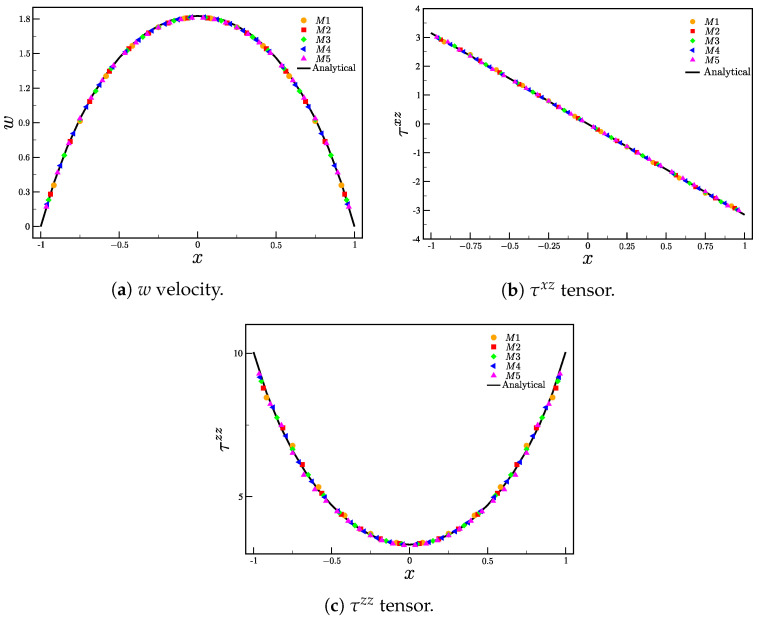
Comparison between the analytical and numerical solutions for (**a**) *w* velocity component and tensors components (**b**) $\tau^{xz}$ and (**c**) $\tau^{zz}$.

Similar to the velocity vector, initial conditions for pressure and tensors are set to zero in full cells. As shown in Figure 4a,b, the pressure and $\tau^{xz}$ tensor profiles are in agreement with the physically expected profiles, i.e., linear profiles across the longitudinal direction (flow direction) and transverse direction (perpendicular to the flow), respectively. In addition, the values obtained for $\tau^{xz}$ are comparable with the behavior of the analytical solution (see Figure 5b). In the cross-section represented in Figure 4b, there is an influence of the inflow (tensors are defined as zero in the inflow, and the Finger tensor is defined as the identity matrix), but, outside this region, the expected linear profile is obtained as previously stated (for further details, refer to Figure 5b).

Figure 5a–c show, respectively, the solution for the velocity *w* and stress components $\tau^{xz}$ and $\tau^{zz}$ using meshes M1–M5. The profiles were obtained at y=0 and were taken in the center of the pipe, $z=z_{max}/2$ for t=10 (or $\bar{t}=25\;s$). The results were compared with the semi-analytical solution, with good agreement between the numerical results (*M*1–*M*5) and the semi-analytical results. The instant t=10 ($\bar{t}=25\;s$) was chosen because the velocity and stresses already show a steady state behavior (the velocity residual is small (see Equation (42)). The residual (for the velocities) is calculated as

(42) $$\;R_{es}=\frac{\sqrt{\sum_{i=1}^{N_{c}}\left\{{\left(u_{i}^{t}-u_{i}^{t-\Delta t}\right)}^{2}+{\left(v_{i}^{t}-v_{i}^{t-\Delta t}\right)}^{2}+{\left(w_{i}^{t}-w_{i}^{t-\Delta t}\right)}^{2}\right\}}}{N_{c}},$$

where *t* is the simulation time, Δt is the time step and $N_{c}$ is the number of computational cells. The slight variances between the analytical and numerical solutions can be attributed to approximations made in the numerical simulations, which differ from the precise analytical solution. Although the tube’s length appears adequate for the complete development of velocity and shear stress profiles, this completeness is not reflected in the $\tau^{zz}$ tensor. Consequently, these disparities remain minor. It is worth noting that, across all meshes, the average relative error remains below 5%.

**Figure 6:**
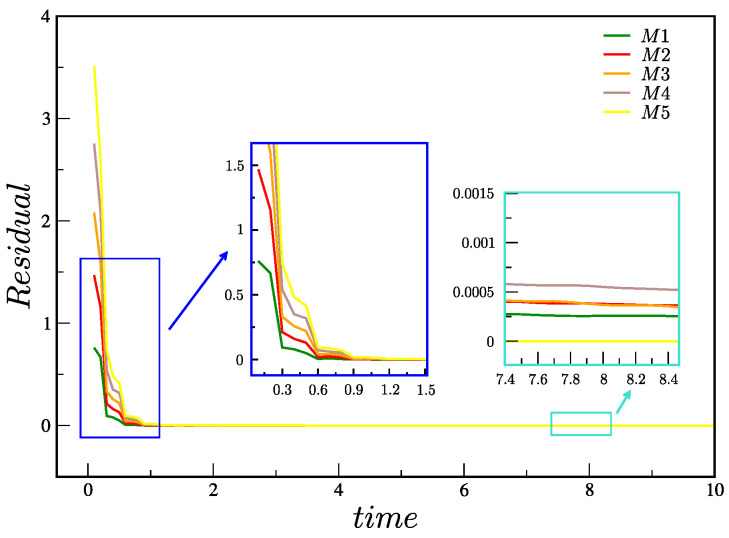
Total residual in meshes M1, M2, M3, M4 and M5 up to t=10 (or $\bar{t}=25\;s$).

Figure 6 shows the calculation of the residuals $R_{es}$ in meshes *M*1–*M*5 up to time t=10 (or, equivalently, $\bar{t}=25\;s$). It can be observed that the residuals in the five meshes *M*1–*M*5 decrease and show convergence towards a steady-state solution, thus proving the robustness of the numerical method. As expected, we also observe a smaller residual for the most refined meshes.

### 5.2. Free Surface Flows

In this subsection, we test the numerical method’s robustness by simulating the extrudate swell phenomenon of Boger fluids. Please note that our aim is not to conduct an in-depth study of this type of flow in Boger fluids; instead, we focus on assessing the reliability of the numerical approach.

**Figure 7:**
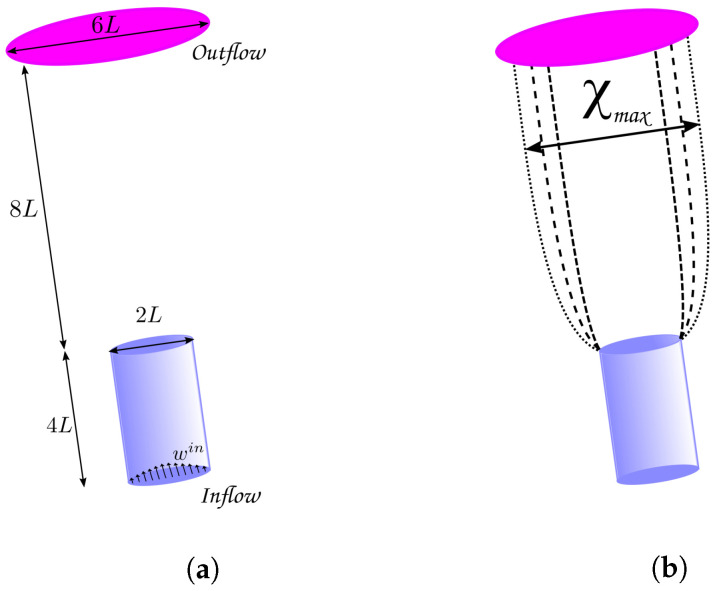
Schematic of a free surface simulation in the FREEFLOW-3D software. (**a**) Schematic representation of the domain; (**b**) illustration of the extrudate swell. The fluid exits the tube and starts to swell.

The phenomenon known as extrudate swell is very present in various industrial processes. In this phenomenon, the fluid flows over a *pipe/die* and swells outside the free surface region (the cross-sectional area of the extrudate—the material being extruded—is larger after exiting the die compared to the die orifice). This behavior is mainly due to the elastic recovery of the polymer chains after being subjected to high pressures and shear forces during the extrusion process. Figure 7a shows the domain used in the simulation, and Figure 7b illustrates the phenomenon of extrudate swell (with the contour lines representing the trajectory of the fluid’s free surface).

It should be remarked that simulating extrudate swell can be challenging due to several numerical difficulties, which are now outlined: the extrusion process involves highly non-uniform and complex flow patterns, especially near the die exit. These flows experience rapid changes in pressure and velocity, making it difficult to model accurately; simulating extrusion processes requires discretizing the computational domain into smaller elements or cells, and the geometry of the die can be quite intricate. Moreover, the simulation must maintain numerical stability, which can be problematic in high-pressure and high-shear regions; simulating extrudate swell is computationally intensive, especially for large-scale industrial extrusion processes and using integral models; extrudate swell is a time-dependent phenomenon as the material continuously deforms and recovers during extrusion. Capturing this transient behavior accurately in numerical simulations requires precise time-stepping algorithms and may increase computational complexity. To address these challenges, researchers often resort to simplifications and assumptions to reduce computational complexity. However, in the context of this work, we take a different approach by considering the complete system of equations and accounting for the full 3D geometry.

To test the robustness of the new numerical procedure, two different Weissenberg numbers were considered (case **C**1 −Wi=0.43 and case **C**2 −Wi=0.64 ), both using non-shear-thinning highly elastic polymer solutions (Boger fluids — see Table 1). Boger fluids are a type of dilute polymer solution known for their remarkable elasticity, particularly at low apparent shear rates, and this unique characteristic gives rise to a significant extrudate swell during the extrusion process [14,36,37,38]. This makes Boger fluids ideal to test the numerical implementation. Numerical simulation of extruded swelling in two dimensions using the data used here (see Table 1) is presented in Mitsoulis [14].

The following parameters were used in the simulations (see Mitsoulis [14] and Tomé et al. [32]):

- Pipe dimension: 0.04m×0.04m×0.08m; $\delta x=\delta y=\frac{0.04}{16}$ (see Figure 7a);
- Pipe diameter 2L=0.04m;
- Number of deformation fields N=50;
- **C**1 − $U=0.1067\;{m.s}^{-1}$, Re=0.7, Wi=0.43;
- **C**2 − $U=0.1600\;{m.s}^{-1}$, Re=1.1, Wi=0.64.

**Figure 8:**
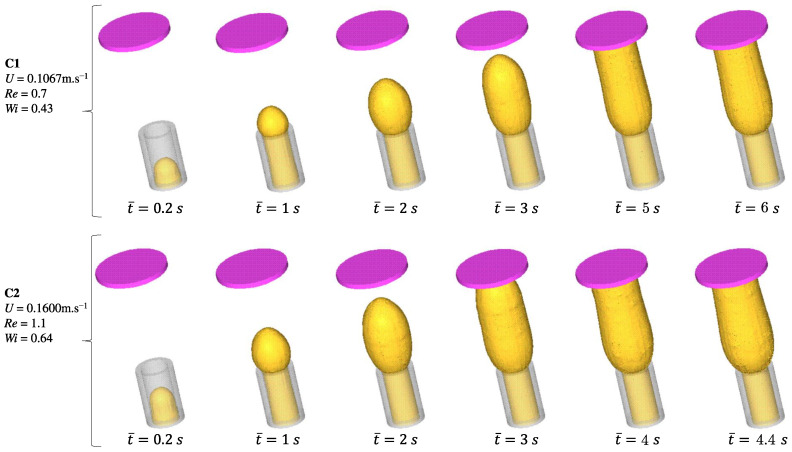
Flow development of a Boger fluid for two different inlet velocities (cases C1 and C2).

Figure 8 shows the flow development of a Boger fluid for two different Weissenberg number values (cases C1 and C2). We conducted flow measurements at six different time points to analyze the behavior of the fluid in the system. The first four time instants were identical for both C1 and C2 cases, while the last two time points differed. Specifically, for the lower inlet velocity case, we considered time points $\bar{t}=5$ and 6 s, and, for the other case, the time points were $\bar{t}=4$ and 4.4 s. During the initial stages of both cases, the fluid exhibited a smooth flow with a parabolic profile as it exited the tube. However, as the process continued, swell occurred, causing the cross-sectional area of the extrudate to increase after leaving the die. This swelling behavior significantly affects the flow dynamics and needs to be carefully considered in the analysis. The simulation results show the development of the fluid front as it reaches the wall. Notably, there are distinct differences in swelling between two specific time instances: $\bar{t}=6\;s$ (C1) and $\bar{t}=4.4\;s$ (C2).

To characterize the extrudate swell phenomenon, an important parameter is the dimensionless swelling rate $\chi =\chi_{\max}/\left(2r\right)$, where $\chi_{\max}$ is the maximum swelling value and *r* is the pipe radius. For case C1, the maximum swelling value is found to be χ=1.78, while, for case C2, the swelling rate increases to χ=1.95. This was expected since the Weissenberg number represents the ratio of the characteristic time scale of the elastic forces acting on a fluid to the characteristic time scale of viscous forces, and, when a polymer melt is subjected to shear flow (for example, in an extruder), the long polymer chains experience deformation due to the flow-induced stretching and alignment. A higher Weissenberg number indicates a more elastic behavior of the polymer melt, leading to more significant elastic recovery and increased extrudate swell.

We may therefore conclude that the numerical method employed in the simulations demonstrates its capability to capture the transient physics of the extrudate swell problem in detail, even for a small difference in the Weissenberg number (cases C1 and C2). It accurately predicts the swelling behavior and allows for a better understanding of the process dynamics. By accounting for the material properties and flow conditions, the simulation provides valuable insights into the extrusion process and contributes to the optimization of extrusion operations.

## 6. Conclusions

In this work, a novel numerical method was developed to address three-dimensional unsteady free surface flows incorporating integral viscoelastic constitutive equations, specifically the K–BKZ–PSM (Kaye–Bernstein, Kearsley, Zapas–Papanastasiou, Scriven, Macosko) model. To implement this new approach, we integrated it into the FREEFLOW-3D code [27], enhancing its capabilities for handling viscoelastic fluid behavior.

To validate the numerical methodology, we conducted simulations of K–BKZ–PSM fluids in a pipe. The results were compared with a newly derived semi-analytical solution, and we found that the simulations performed on five different meshes yielded excellent agreement with the analytical solution. Furthermore, we applied our methodology to tackle flows with free surfaces. One notable example was the simulation of the classic extrudate swell problem, which involved a highly elastic polymeric solution known as the Boger fluid.

The significance of this work lies in the scarcity of literature concerning the simulation of unsteady three-dimensional flows of K–BKZ–PSM fluids (and integral viscoelastic models in general) using finite differences, especially when considering problems with moving free surfaces. Therefore, we hope that our contributions will inspire and encourage other researchers to further develop and explore the numerical methods we have presented here.

In conclusion, our newly developed numerical method has proven its effectiveness in handling complex fluid flow scenarios, including free surface flows such as extrudate swell simulations. The successful validation against analytical solutions reinforces the reliability of our approach and opens up opportunities for broader applications in the field of viscoelastic fluid dynamics.

## Figures and Tables

**Table 1:** Parameters of the fluid used in the extrudate swell problem (see Mitsoulis [14]).

$\rho_{0}=868\;{\mathrm{kg}/m}^{3}$, α = 34,214			
$\lambda_{ref}=0.081$ s, β=0.1, $\eta_{0}=2.4$ Pa.s			
*k*	$\lambda_{k}$ [s]	$a_{k}$ [Pa]	$\eta_{k}$ [Pa.s]
1	$0.4887\times 10^{-3}$ s	$3.1295\times 10^{3}$ Pa	1.5294 Pa.s
2	$0.4464\times 10^{-1}$ s	$5.0917\times 10^{0}$ Pa	0.2273 Pa.s
3	$2.8384\times 10^{-1}$ s	$2.2783\times 10^{0}$ Pa	0.6457 Pa.s

## Data Availability

Not applicable.

## Abbreviations

The following abbreviations are used in this manuscript:

K–BKZ	Kaye–Bernstein, Kearsley, Zapas
PSM	Papanastasiou, Scriven, Macosko
EVSS	Elastic–Viscous Split Stress
UCM	Upper-convected Maxwell
PTT	Phan–Thien–Tanner
FENE-P	Finite Extensible Nonlinear Elastic Peterlin
HDPE	High-Density Polyethylene
LDPE	Low-Density Polyethylene
FEM	Finite element Method
FDM	Finite Difference Method
PDE	Partial Differential Equation
ALE	Arbitrary Lagrangian Eulerian
FBC	Free Boundary Condition
